# Small hydrophobic viral proteins involved in intercellular movement of diverse plant virus genomes

**DOI:** 10.3934/microbiol.2020019

**Published:** 2020-09-21

**Authors:** Sergey Y. Morozov, Andrey G. Solovyev

**Affiliations:** 1 A. N. Belozersky Institute of Physico-Chemical Biology, Moscow State University, Moscow, Russia; 2 Department of Virology, Biological Faculty, Moscow State University, Moscow, Russia; 3 Institute of Molecular Medicine, Sechenov First Moscow State Medical University, Moscow, Russia

**Keywords:** plant virus, intercellular movement, hydrophobic protein, trans-membrane domain, evolution, triple gene block, RNA genome, DNA genome, protein motifs

## Abstract

Most plant viruses code for movement proteins (MPs) targeting plasmodesmata to enable cell-to-cell and systemic spread in infected plants. Small membrane-embedded MPs have been first identified in two viral transport gene modules, triple gene block (TGB) coding for an RNA-binding helicase TGB1 and two small hydrophobic proteins TGB2 and TGB3 and double gene block (DGB) encoding two small polypeptides representing an RNA-binding protein and a membrane protein. These findings indicated that movement gene modules composed of two or more cistrons may encode the nucleic acid-binding protein and at least one membrane-bound movement protein. The same rule was revealed for small DNA-containing plant viruses, namely, viruses belonging to genus *Mastrevirus* (family *Geminiviridae*) and the family *Nanoviridae*. In multi-component transport modules the nucleic acid-binding MP can be viral capsid protein(s), as in RNA-containing viruses of the families *Closteroviridae* and *Potyviridae*. However, membrane proteins are always found among MPs of these multicomponent viral transport systems. Moreover, it was found that small membrane MPs encoded by many viruses can be involved in coupling viral replication and cell-to-cell movement. Currently, the studies of evolutionary origin and functioning of small membrane MPs is regarded as an important pre-requisite for understanding of the evolution of the existing plant virus transport systems. This paper represents the first comprehensive review which describes the whole diversity of small membrane MPs and presents the current views on their role in plant virus movement.

## 1. Introduction

As plant cells contain a tough, rigid cell wall, virus cell-to-cell movement occurs through plasmodesmata (PD), the channels in cell walls interconnecting neighboring cells. In the PD pore, the plasma membrane (PM) is continuous between the two cells, and the endoplasmic reticulum (ER) traverses the pore as a tightly appressed tube called the desmotubule [1]–[7]. It has been suggested that filaments composed at least partly from actin are located in the PD cytoplasmic sleeve between PM and desmotubule [3]–[6]. The size exclusion limit (SEL) of the unmodified PD channel is near 1000 daltons, thus allowing intercellular passage of only small molecules. The size of virions or virus-specific ribonucleoproteins (RNPs) (>10 nm) exceeds the PD SEL [3],[8]–[11]. Thus, modification of PD SEL by plant viruses is absolutely required for their movement from cell to cell. To perform their intercellular transport *via* Pd, viruses have evolved to encode specialized transport systems that usurp pre-existing host pathways for symplastic macromolecular communication in plants [12]–[18]. Retrospectively, over 40 years have passed since the hypotheses were put forward that consider the virus movement in plants as an active process used by plant viruses (particularly, tobacco mosaic virus) to spread both locally and systemically throughout plant bodies with the aid of specialized movement proteins (MPs, or transport proteins-TPs) [19]–[22]. The first evidences suggesting that cell-to-cell movement is controlled by a single movement protein have been provided by studies of a 30-kDa protein of *Tobacco mosaic virus* (TMV) [23]–[26]. It has been found that this MP can alter the size exclusion limit of PD and bind ssRNAs, and may move through PD as an RNP complex comprising virus genomic RNA. Some MPs in single MP-encoding viruses, similar to the TMV 30-kDa protein, possess all required protein domains (motifs) to perform cell-to-cell movement independently from other virus-specific proteins. Moreover, such single MP-encoding viruses are generally capable of sequence non-specific RNA binding and can assist the movement of other transport-deficient viruses [9],[12],[18],[27]–[29].

However, there is a special protein class of ‘30-kDa’-like MPs [30], which use an alternative mechanism for cell-to-cell transport. This mechanism includes the displacement of the Pd internal structures including the desmotubule by a tubular structure formed by viral MP and the transfer of whole virions inside the tubule from infected cells to neighboring cells [9],[12],[18],[31]. Virions can pass Pd inside MP-formed tubule in some positive strand RNA viruses, namely, comoviruses and nepoviruses (family *Secoviridae*) [31],[32], and pararetroviruses [18],[33]–[37]. Analogously, plant negative strand RNA viruses representing families *Tospoviridae* and *Aspiviridae* encode ‘30-kDa’-like MP performing tubule-guided cell-to-cell movement of capsid RNPs [38]–[41].

**Figure 1.**
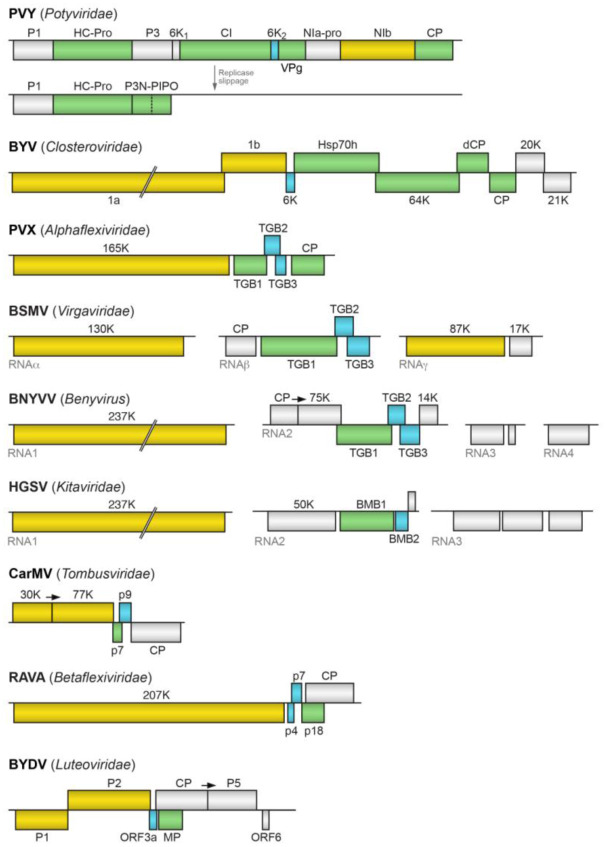
Comparison of plant virus RNA genomes encoding multicomponent cell-to-cell transport systems. Genes are shown as boxes with the names of the encoded proteins. Genes of proteins involved in cell-to-cell movement are shown in green. Genes encoding small hydrophobic proteins are shown in blue. Replicative genes are shown in yellow. Arrows indicate read-through codons. PVY, *Potato virus Y*; BYV, *Beet yellows virus*; PVX, *Potato virus X*; BSMV, *Barley stripe mosaic virus*; BNYVV, *Beet necrotic yellow vein virus*; HGSV, *Hibiscus green spot virus*; CarMV, *Carnation mottle virus*; RAVA, *Ribes americanum virus A*; BYDV, Barley yellow dwarf virus.

A number of taxonomic groups of plant RNA viruses encode several MPs, acting in concert, to move viral genomes from cell to cell [18]. Viruses belonging to families *Potyviridae* [42]–[44] and *Closteroviridae* [45]–[48] are shown to have several proteins functioning together with capsid proteins (CPs) to enable virus cell-to-cell movement in infected plants (Figure 1) (see below for more details). Other functionally similar MPs are encoded by evolutionarily conserved gene modules including double gene block (DGB), triple gene block (TGB) and binary movement block (BMB) [18],[49]–[58]. DGB and BMB encode two proteins, whereas TGB codes for three proteins.

Among the above mentioned plant virus movement systems, which do not depend on the ‘30-kDa’-like MPs, all encode one or two small hydrophobic proteins comprising vital components of virus movement machinery. Here we review the latest discoveries in relation to the small hydrophobic MPs. Specifically, this review will discuss common sequence properties of these proteins related to their movement functions and evolutionary aspects of their origination and adaptation to the role in viral movement systems. We also discuss some novel discoveries of additional potential examples of putative small hydrophobic MPs.

## 2. Potyviruses

### 2.1. Proteins of potyviral movement system

The best-studied genus of the family *Potyviridae, Potyvirus*, includes viruses with monopartite plus-RNA genome (ca. 10–11 Kb size), which, after generation of two alternative viral RNA templates by transcriptional slippage with a +1 A insertion in the motif GAAAAA, produces two overlapping polypeptides. After proteolytic processing by three viral-encoded proteinases, the main polyprotein gives rise to ten proteins, which include P1, HC-Pro (helper component proteinase), P3, 6K1, CI (cylindrical inclusion protein), 6K2, NIa (nuclear inclusion protein with two parts, genome-linked protein VPg and protease), NIb (large nuclear inclusion protein with the function of RNA-dependent RNA polymerase, RdRp), and capsid protein (CP) (Figure 1). Notably, the number of proteins produced after polyprotein cleavage may vary in other genera of *Potyviridae*. The smaller polyprotein in genus *Potyvirus* include P1, HC-Pro and P3N-PIPO (Pretty Interesting Potyviridae ORF) [43],[58]–[65].

During infection, RNA viruses re-organize cell membranes into ‘viral factories’ that are intracellular virus-specific compartments serving as sites of virus replication. These virus replication compartments (VRCs) contain viral RNA as well as virus and host proteins involved in genomic RNA replication [66]–[72]. In potyviruses, almost all virus polypeptides forming VRCs are involved in intercellular RNA genome movement as well. The VRC vesicles represent remodeled ER membranes serving as the membranous scaffold for potyvirus RNA replication. In potyviruses, a small viral membrane protein 6K2 is involved in the local spatial re-arrangement of the ER membrane and the formation of vesicular VRC at the ER exit sites [59],[65],[73]–[76]. Several other viral proteins including CI (replicative SF2 RNA helicase), 6K1, 6K2, NIa, HC-Pro, P3, and P3N-PIPO have been shown to be functional protein elements of VRC in addition to NIb [65],[72],[77],[78]. The VRC with incorporated viral CP may work as the motile vesicles moving along actin filaments and involved in virus replication, the genome intercellular movement and localization on chloroplasts [42],[65],[76],[79],[80]. Moreover, 6K2, VPg, CP, and CI proteins are known to interact with *Arabidopsis thaliana* dynamin-like protein AtDRP2 [81], which belongs to a protein family playing an essential role in membrane re-modelling and fusion, therefore it is possible that the potyviruses may use co-optation of AtDRP2 and 6K2 for VRC assembly.

The N-terminal cytoplasmic tail of 6K2 is important for the interaction, *via* a conserved di-acidic D(X)E motif, with the COPII subunit Sec24A and further ER export of VRC [75],[82]. As an additional factor, which may influence VRC vesicle formation on the ER membrane, P3 can bind a reticulon-like protein known to re-shape ER membranes [83]. Further movement of VRC to cell periphery and PD requires a nonconventional pathway bypassing ER-Golgi trafficking and involving VTI11, a pre-vacuolar compartment SNARE protein [84]. P3N-PIPO, which binds to VRC through P3-P3N interactions [72], is a critical component of VRC vesicles specifically required for the PD targeting, and the PD association of P3N-PIPO depends on the PIPO domain [85].

As revealed by ultrastructural analyses, the CI protein is associated with the formation of cone-shaped structures, which are localized at the PD entrance and include also CP and viral RNA [86], and the PD localization of CI has been shown to depend on its interaction with P3N-PIPO [79]. These inclusion bodies at structurally modified PDs may serve as docking and conducting structures coordinating different viral proteins in the intercellular movement of viral replication vesicles and virions. In addition, VPg also may positively influence cell-to-cell potyvirus movement inducing proteasome-mediated degradation of plant protein remorin, thus preventing formation of remorin-specific plasma membrane nanodomains, which reduce membrane plasticity, and subsequent PD closure [5]–[7],[44],[60],[86]–[90].

6K2-induced vesicles are the main containers involved in cell-to-cell and long-distance systemic movement of potyvirus genomic RNAs [42],[72],[80],[91],[92]. It is still debated how VRC vesicles can be used for systemic transport through the conducting elements of the phloem. It is possible that individual vesicles in Pd between companion cells and sieve elements can merge and form large membrane complexes that are stationary, but are able to produce large amounts of genomic RNA, which is used in the presence of a capsid protein for assembly of virions. The latter are then released to the conducting elements for long distance transport [80],[92].

Unexpectedly, experimental evidences suggest the possibility of movement of potyvirus replicative vesicles in xylem vessels. The vesicles may enter the protoxylem by intercellular transport to immature cells of the xylem elements, where the virus replicates in the cytoplasm before programmed cell death occurs and the xylem becomes a hollow vessel; then vesicles may travel long distances *via* the xylem network [92]. Another novel striking route of potyvirus movement within plant bodies is suggested by recently discovered extracellular VRC location, which is preceded by fusion of VRC aggregates with the plasma membrane and requires plant protein VTI11 [76],[84].

### 2.2. 6K2 transmembrane domain and membrane re-modelling

In many members of genus *Potyvirus*, 6K2 protein is 53–54 amino acids long [60], and its transmembrane domain encompasses amino acids 25–43 (19 residues in length) (Table 1). This transmembrane domain overlaps alpha-helices H2 and H3 (H2 correspond to residues 15–32, and H3–to residues 36–45) [93],[94]. Importantly, 6K2 protein can induce the formation of VRC-like vesicles even when it is transiently expressed in plants in the absence of other viral proteins [44],[76],[93],[95]. The transmembrane domain (TMD) of 6K2 contains a well-known GxxxG motif of protein membrane-bound domains, mutations of which disturb intracellular localization of 6K2 and inhibit virus replication as well as cell-to-cell movement [84],[93]. This motif is conserved in the members of several genera among *Potyviridae* (Table 1). Generally, it was shown that TMD helices of different membrane proteins in animals and plants can mediate protein hetero- and self-oligomerization involving intramembrane close inter-helical contacts and carbon-hydrogen bond formation inside GxxxG and GxxxG-like sequence motifs (GxxxG, GxxxA, SxxxG, etc.) consisting of small amino acids (Gly, Ala, and Ser) with intercalating any three amino acids (Table 1) [96]–[99].

In view of these data, it can be proposed that functional VRC vesicles are formed due to intra-ER oligomerization of 6K2 itself, or because of 6K2 interactions with some plant membrane-bound proteins. Recent data, however, strongly suggest that the main role of the 6K2 GxxxG-like motif can involve interaction with plant dynamin-like proteins of ROOT HAIR DEFECTIVE3 (RHD3) family (including AtDRP2), as trans-membrane segments of these proteins also may contain GxxxG-like motifs [76],[84],[94],[99]. Dynamins represent one of the major protein components of eukaryotic cells involved in membrane re-shaping [6],[100].

**Table 1.**
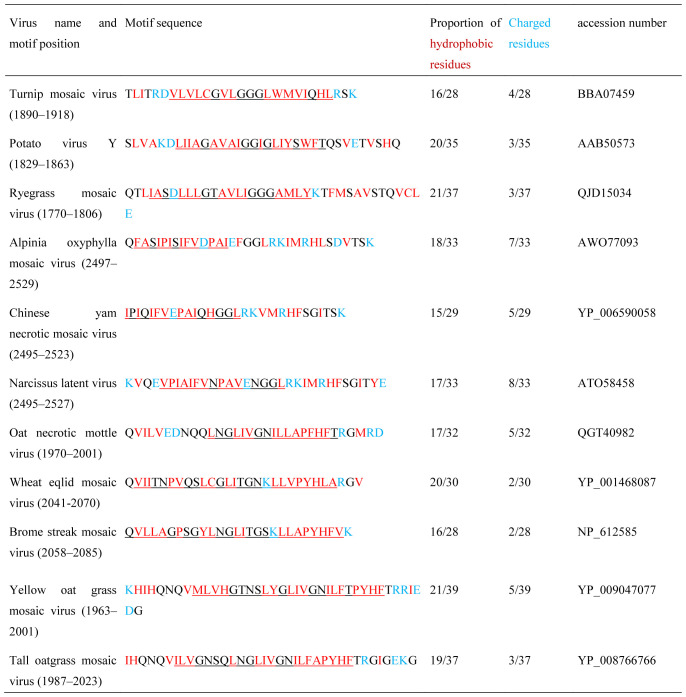
Hydrophobic motif comparisons among the small 6K2 proteins encoded by some virus genomes in family *Potyviridae*.

Motif position means coordinates of the sequences presented in the table, and proportions are related only to the sequences included into the table. GxxxG-like sequence motifs are shown in yellow. Transmembrane segments predicted by (http://www.cbs.dtu.dk/services/ TMHMM-2.0/) are underlined. Note that A, H, P and C residues are often regarded as hydrophobic in TMD segments.

## 3. Closteroviruses

The plant viruses belonging to the family *Closteroviridae* may encode a dozen of proteins and possess very large positive-stranded RNA genomes (near 20 kilobases in length), which could be compared only with those of animal nidoviruses [101]–[105] whose genomes, however, can reach an unprecedented size of more than 41 kilobases, as it has been found in planarian nidovirus [106]. This virus family includes four genera: *Closterovirus*, *Velarivirus*, *Ampelovirus* and *Crinivirus*. The latter genus is represented by bi- and tripartite viruses in contrast to other monopartite closteroviruses [103],[105]. In genus *Closterovirus*, five genes represent a unique gene module found to participate in virus intercellular movement and named quintuple gene block (QGB) [101],[107]. QGB codes for (from 5′ to 3′) a ∼6-kDa hydrophobic protein (p6), an Hsp70 homologue (Hsp70h), a ∼60-kDa protein (p60), the minor capsid protein (CPm), and the major capsid protein (CP) (Figure 1). Variations of QGB in different closteroviruses include duplication, shuffling and divergence of the CP and CPm genes, as well as acquisition of additional ORFs within QGB of criniviruses and certain ampeloviruses [102],[103],[105],[108]–[110].

CP coats ∼95% of the viral RNA, and assembly results in formation of a long helical flexuous virus particles. The remaining 5% of the RNA genome contains a packaging signal recognized by CPm, and the resulting ‘rattlesnake structure’ represents an unusual particle architecture with a short virion tail, which can be assembled independently of the main virion body [101],[103],[110]. The tail assembly requires Hsp70h and p60 proteins, which represent minor tail components and act cooperatively to facilitate incorporation of CPm. Like cellular chaperones of the Hsp70 family, closteroviral Hsp70h possesses a highly conserved, N-terminal ATPase domain, whereas p60 contains the C-terminal domain close to the closterovirus CP and CPm. Moreover, this domain is conserved in almost all capsid proteins of filamentous viruses, thus suggesting that p60 may directly interact with RNA [101]–[103],[110],[111]. It has been proposed that the tail of closteroviral virions evolved to facilitate cell-to-cell and systemic transport of the large closterovirus encapsidated genomic RNAs [111],[112]. Indeed, virions and Hsp70h were found in PD [112],[113]. It is important that each of the four structural proteins of the closteroviral virions is also required for virus movement [110],[111].

Hydrophobic p6 is a single-span transmembrane protein that functions in virus cell-to-cell movement. Because p6 is not required for virus replication or assembly, it is regarded an MP [102],[103],[114]. Analogs of p6 in genera *Closterovirus* and *Crinivirus* possesses certain common features found in a number of small hydrophobic movement proteins of other plant viruses: these small proteins localize to the ER and Pd, and are capable to influence Pd size exclusion limit [102],[103],[114]–[118]. Despite probable common function, p6 analogs throughout members of the family *Closteroviridae* are not well conserved (Table S1). It should be noted that some members of genus *Crinivirus* show remarkable deviations connected to peculiarities of p6 coding details. First, tripartite genome of *Potato yellow vein virus* contains the gene for the p6 analog in the 3′-terminal region of RNA1 but not in RNA2 as it was found for other criniviruses [119]. Second, the 5′-terminal region of *Lettuce chlorosis virus* genome contains two short genes coding for hydrophobic proteins, thus it is not clear whether one or both these proteins are required for virus cell-to-cell movement [120],[121].

## 4. Triple gene block

Many positive-stranded RNA plant viruses contain a conserved module of three overlapping genes termed ‘triple gene block’ (TGB). Comparative genome sequence analysis revealed that the TGB is present in a large number of viruses belonging to families *Alphaflexiviridae* and *Betaflexiviridae* (genera *Potexvirus*, *Allexivirus*, *Mandarivirus*, *Lolavirus*, *Foveavirus*, *Carlavirus* and *Robigovirus*) [122]–[124], *Virgaviridae* (genera *Hordeivirus*, *Pomovirus*, *Goravirus* and *Pecluvirus*) [125]–[127] and genus *Benyvirus* [128]. The three TGB-encoded proteins referred to as TGB1, TGB2 and TGB3 act in concert to deliver viral genomes to and through PD into adjacent cells. Interestingly, many genomes of viruses belonging to the genus *Allexivirus* includes a TGB3-like protein-encoding sequence lacking an AUG initiator codon. This TGB3 gene is translated from a non-AUG initiator codon [129]. Two cassava-infecting viruses of the genus *Potexvirus* (family *Alphaflexiviridae*), namely *Cassava virus X* (CsVX) and *Cassava new alphaflexivirus* (CsNAV) having a gene arrangement typical for potexviruses, but lack the TGB3 gene [130]. CsVX is rather inefficiently transmitted to *Nicotiana benthamiana*, whereas CsNAV causes no infection of this plant host [130]. These data support our earlier hypothesis [129],[131],[132] that a TGB3-related gene could be an accessory, rather than essential, TGB component, which can be necessary in certain hosts species or plant tissues at least in potexviruses [133],[134].

TGB1 contains the domain of an RNA helicase of superfamily 1 (SF1), whereas TGB2 and TGB3 represent small membrane-associated proteins [18],[29],[52],[54]. The TGB modules are subdivided into several different groups based on the structural properties of the encoded proteins. TGB3 proteins form two distinct groups, potex-like and hordei-like TGBs, depending on the presence of a single (genera *Potexvirus*, *Carlavirus*, *Foveavirus* and *Allexivirus*) or two (genera *Hordeivirus*, *Pomovirus*, *Goravirus* and *Pecluvirus*) TMDs (Tables S2 and S3). Recently, we have revealed two additional TGB classes, one of which combines TGBs of several related viruses belonging to the genus *Benyvirus* (see below), whereas another one is represented by the TGB of virus-like RNA assembly in the transcriptome of *Colobanthus quitensis* (Cq-VLRA) resembling a plant virus genome fragment encoding proteins distantly related to the respective proteins of the genus *Benyvirus* [131],[132] (Table S4). Like the hordei-like TGB3 proteins, the benyvirus TGB3 has two transmembrane domains; however, the benyvirus TGB3 protein differs from hordei-like proteins by the location of N-terminal transmembrane domain very close to the protein terminus and a conserved sequence signature found only in the genus *Benyvirus* [52],[131],[132],[135] (Table S4). In Cq-VLRA, the central hydrophilic region of Cq-VLRA TGB3 protein exhibits conservation of most amino acid residues found to be invariant in TGB2 proteins, and we have proposed that Cq-VLRA encodes an evolutionary early variant of TGB [131]. Generally, it is accepted that TGBp2 and TGBp3 proteins are involved in both homologous and heterologous protein-protein interactions and can be involved in TGBp1 targeting to PD [136]–[138].

### 4.1. Alphaflexiviridae and Betaflexiviridae

The genome organization of viruses belonging to families *Alphaflexiviridae* and *Betaflexiviridae* are quite similar ) [122]–[124] (Figure 1). Accordingly, two different families have been found to use similarly organized TGB proteins and likely share the cell-to-cell transport mechanism, which has been extensively studied for Potato virus X (PVX, family *Alphaflexiviridae*). Early in potexvirus infection, TGB2 is incorporated into ER-derived membranous vesicles that possibly represent early VRCs and contain replicase, TGB1 and TGB3 [3],[54],[139]–[143]. After replication, VRCs may travel to the cellular periphery along the ER-actin network. Some VRCs anchor at the PD entry where progeny RNA chains may be encapsidated by synthesized CP. These newly formed virions may interact with TGB1; and this complex can be delivered to and through PD with the aid of TGB2 and/or TGB3 [3],[54],[56],[143]–[145]. Accordingly, the potexvirus virions were shown to reside inside PD [146]. Importantly, TGB1 is capable of destabilizing potexvirus virions and might act as a factor that enables translation of virion-derived RNAs after the virus has moved from cell to cell [112],[147],[148].

The potexvirus TGB1 is a multifunctional protein. TGB1 possesses an RNA helicase activity, and contains a set of canonical SF1 helicase motifs that are necessary to unwind double-stranded RNA [54],[149],[150]. In addition to RNA helicase activity, the TGB1 proteins may participate in gating Pd [133],[151],[152]. Additionally, TGB1 proteins of *Alphaflexiviridae* and *Betaflexiviridae* have been shown to act as suppressors of RNA silencing. Mutations that inhibited silencing suppressor activity also blocked virus cell-to-cell movement, suggesting that silencing suppression could be linked to virus transport [142],[153]–[156].

It should be noted that previously distinguished two types of TGBs, namely potex-like and hordei-like TGBs, have similarly organized TGB2 proteins with two TMDs (Tables S2 and S3) and a highly conserved central region between them in contrast to more significantly diverged TGB1 and TGB3 [52],[54],[132]. We have suggested that the TGB3 cistron could emerge in the transport gene module including TGB1 and TGB2 as a result of horizontal gene transfer, and/or duplication of the TGB2 gene and subsequent divergence of the two TGB3 gene lineages with a single or two TMDs [131].

### 4.2. Virgaviridae

Most studies of hordei-like TGB movement systems were concentrated on genera *Hordeivirus* and *Pomovirus* [18],[29],[54],[157],[158] (Figure 1). In contrast to viruses with potex-like TGB, the viral movement mediated by the hordei-like TGB does not require no the viral CP [54]. As an alternative to the virion as a genome transport form of viruses with potex-like TGB, the RNAs of viruses with hordei-like TGB form RNP complexes with TGB1 [159]–[161]. In hordei-like TGBs, the TGB3 interacts with TGB2 and the TGB1/RNA complexes to form a movement-competent form of virus genome that is directed to PD in connection with the ER [18],[127],[137],[138],[158]. It has been suggested that TGB2 and TGB3 proteins may be recycled from PD-associated sites to the cytoplasm by endocytic vesicles [18],[127],[158].

Hordei-like TGBs have similarly organized TGB2 and TGB3 proteins, each of which has two TMDs (Table S3) and a highly conserved central region between them [52],[54],[132]. Hordeiviral TGB3 possesses an oligomerization signal, and its C-terminal TMD has been shown to contain a targeting motif responsible for TGB3 trafficking to PD-associated membrane compartments [162]. TGB2 in hordei- and pomoviruses is found either to be essential for TGB3-mediated transport of TGB1 to PD-associated sites, or to increase the efficiency of such transport. The intracellular transport of TGB3 most probably uses a lateral diffusion in the ER membranes [18],[126],[127],[137],[138],[157],[159],[163].

### 4.3. Family Benyviridae and Nicotiana velutina mosaic virus

RNA1 of benyviruses contains one large ORF coding for a replication-associated protein that includes methyltransferase (MET) motif in the N-terminal part, helicase (HEL) and papain-like protease motifs (PROT) in the central part, and RdRp motif in the C-terminal part (Figure 1). RNA2 of a typical benyvirus exemplified by that of Beet necrotic yellow vein virus (BNYVV) possesses six ORFs; namely, the CP gene terminated by a suppressible stop codon, the CP readthrough protein gene, the TGB coding for TGB1, TGB2 and TGB3 and a cistron coding for the cysteine-rich protein having the silencing suppressor activity (Figure 1) [128],[164],[165]. The N-terminal region of the benyvirus TGB1 has nucleic acid binding activity and contains consensus sequence motifs characteristic of an ATP/GTP-dependent SF1 helicase [165]. The three TGB proteins are found in the peripheral membrane bodies that seem to be derived from ER; TGB1 is targeted by TGB2 and TGB3 to peripheral punctate bodies associated with PD [128],[165].

Unassigned RNA virus *Nicotiana velutina mosaic virus* (NVMV) has a genome consisting of RNA1 (8 Kb) and RNA2 (3 Kb), which are encapsidated in rigid, rod-shaped particles [166]. The RNA2-encoded ORF1 encodes a protein showing relatively weak but significant similarity to coat proteins (CPs) of viruses of the genus *Benyvirus* whereas the ORF2 protein was found to have closest similarity to benyvirus TGB1 helicases [52],[132],[166]. NVMV ORF3 protein is a TGB2 protein with two hydrophobic regions and hydrophilic signature in the central part, which is highly similar to that of benyvirus TGB2 (Table S4). The NVMV ORF4-encoded protein has the benyvirus-like TGB3 organization with two hydrophobic regions at N- and C-termini [131],[132] (Table S4).

## 5. Binary movement block

A novel transport gene module consisting of two genes and termed ‘binary movement block’ (BMB) has been found in the genome of *Hibiscus green spot virus* (HGSV, genus *Higrevirus*, family *Kitaviridae*) [58],[167]. BMB2 protein is a small integral ER protein and capable of trafficking to Pd-associated sites associated with the formation of ER-derived bodies at the cell periphery. Moreover, BMB2 directs BMB1, having helicase activity, to the membrane bodies, to the Pd interior cavity and to neighboring cells [58]. The BMB2 intracellular transport to Pd-associated sites does not involve the secretory pathway, but requires the functional ER/actin network [168].

HGSV RNA1 has a single ORF encoding the replicase protein, which contains MET, PROT, SF1 HEL and POL domains (Figure 1). This replicative protein shows significant similarity to replicases of plant cileviruses, furoviruses, and pomoviruses as well as insect negeviruses [167],[169]. The HGSV BMB1 helicase shows evident similarity to the SF-I replicative helicases of the genus *Benyvirus* [132]. HGSV BMB2 is distantly related to TGB2 proteins and contains two long hydrophobic segments [131] (Table S4).

Our recent data revealed significant similarity to two BMB-like proteins in polypeptides encoded by three long virus-like RNA assemblies (VLRAs) of dicot plants *Lathyrus sativus* (7970 nucleotides), *Quercus castanea* (7776 nucleotides) and *Litchi chinensis* (7388 nucleotides) [131],[132] (Table S4). It should be noted that three revealed VLRAs show a different, compared to HGSV, type of genome organization (monopartite *vs* multipartite). Similarity of HGSV BMB1 helicase to the SF-I replicative helicases of the genus *Benyvirus* as well as relationship of BMB2 to TGB2 proteins suggests that the important step of the TGB evolution could be the acquisition of TGB2-like protein and formation of a distinct, non-replicative, gene module with an autonomized helicase domain from replicative polypeptide [132],[135].

## 6. Double gene block

Cell-to-cell movement of many viruses belonging to the family *Tombusviridae* requires the coordinated actions of two small proteins encoded in the central region of their genomes (Figure 1) [18],[53],[57],[170]–[173]. After TGB, this gene block is referred to as the double gene block (DGB) [51]. Most genera belong to the subfamily *Procedovirinae*, all of which express their RdRps *via* translational readthrough of a stop codon in the 5′-terminal ORF (Figure 1). DGB was found among members of the genera *Alphacarmovirus*, *Alphanecrovirus*, *Betacarmovirus*, *Betanecrovirus*, *Gallantivirus*, *Gammacarmovirus*, *Macanavirus*, *Machlomovirus*, *Panicovirus*, and *Pelarspovirus* [173]. Among proteins encoded by these viruses, only the DGB proteins, DGB1 and DGB2, are required for the genome movement from cell to cell [170]. Carmovirus DGB1s are characterized by the presence of a basic central region with an alpha-helix fold shown to be involved in RNA binding [18],[53],[174],[175], whereas motifs at the DGB1 C-terminus could be involved in interaction with host proteins and self-interaction [176],[177]. The above activities of DGB1 are shown to be directly associated with cell-to-cell movement. This protein is suggested to form movement-competent RNP complexes with genomic RNA that moved along microfilaments and concentrated at the cell periphery in sites located close to PD [18],[177].

The second MP in carmoviruses, DGB2, has been classified into two groups having one or two potential transmembrane domains (TMD) (Table S5) [18],[178]. DGB2 proteins can be co-translationally inserted *in vitro* into ER-derived microsomes and participate in a signal recognition particle-dependent and translocon-assisted processes [18],[57],[178]–[181]. The membrane topology of these proteins has been determined both *in vitro* and *in vivo* [18],[179],[180],[182]. Detailed *in vivo* studies have confirmed that DGB2 proteins associate with plant ER membranes, whereas *Melon necrotic spot virus* DGB2 has been shown to be targeted to PD *via* the Golgi apparatus in a COPII-dependent pathway [171],[180],[182].

DGB2 proteins contain TMDs of about 19–20 amino acids in length (Table S5) crossing lipid bilayer in a single pass, and their ER export depends on specific sorting motifs located in both the cytoplasmic and luminal DGB2 domains [18]. A lateral diffusion of DGB2 along the ER membranes, probably driven by the ER-associated actin system, is required for the protein ER export [178]. The initially proposed model for carmovirus movement implies that the DGB2 cytosolic region can interact with DGB1/RNA complex, and this ternary complex is transported from the replication sites to PD through the endomembrane system [18],[57]. However, some DGB1 proteins have a nuclear localization mediated by two nuclear localization signals that are necessary for virus cell-to-cell movement [18],[57],[176]. Thus, it seems that the mechanisms of the carmovirus cell-to-cell movement could be more complex and, possibly, different models should be considered for distinct viruses.

## 7. Ribes americanum virus A

A novel virus named *Ribes americanum virus A* (RAVA) has been recently found. Its genome organization resembles viruses from family *Betaflexiviridae* [183]. The RAVA genome consists of 7106 nucleotides and includes the poly(A) tail. Five ORFs were identified in the sequence of RAVA genome (Figure 1). The 5′-terminal ORF codes for a betaflexivirus-related replicase that contains a methyl transferase, AlkB (a family of specific demethylases), RNA helicase and RdRp domains. Other 3′-proximal ORFs code for four proteins that exhibit no significant homology to other virus proteins [183]. However, the arrangement of the genes downstream of the first ORF formally resembles that in members of the family *Betaflexiviridae*. Nevertheless, it shows an unconventional positioning of two small ORFs encoding hydrophobic proteins just after replicase-coding ORF, and these small ORFs are followed by two additional cistrons coding for hydrophilic polypeptides (Figure 1). It was predicted that these four ORFs encode three MPs and the viral CP [183]. Our TBLASTn searches for sequences closely related to the RAVA replicase suggest that a VLRA (accession GFCU01057973) found in the NCBI transcriptome database of dicot plant *Rhododendron delavayi* encodes a novel virus evolutionarily close to RAVA (Table S5), suggesting that the RAVA-specific gene arrangement is not unique for this particular virus.

## 8. Luteoviruses

Most viruses in the family *Luteoviridae* belong to genera *Luteovirus* and *Polerovirus*. These viruses, which have monopartite positive-stranded RNA genomes of 5.3 to 5.9kb, are limited to the phloem in infected plants. Genomic RNAs contain 5–7 ORFs (Figure 1) that are expressed by frameshifting, leaky scanning, and termination codon readthrough [184]–[188]. Importantly, luteoviruses and poleroviruses share a gene module, which encodes the CP (ORF3), MP (ORF4 nested into ORF3), and a carboxy-terminal extension to the CP (ORF5) (Figure 1). These three proteins participate in the phloem-specific movement of the viruses in plants and are translated from one subgenomic RNA [184]–[187]. Recently, it has been reported that this subgenomic RNA contains a novel 5′-proximal short ORF, termed ORF3a [189],[190] Importantly, translation of this short ORF starts with non-AUG codons, as it has been previously found for some other plant virus genes, for example, TGB3 gene in allexiviruses [129],[188]. Functional analysis of the ORF3a protein showed its involvement in virus movement through PD when this small protein works in concert with MP [189]–[192]. Importantly, all 3a proteins include long hydrophobic TMD (Table S5), so that the organization of movement gene block (not counting CP gene) is formally similar to DGB and includes single hydrophilic protein and single small hydrophobic protein (see above).

## 9. DNA-containing plant viruses

**Figure 2.**
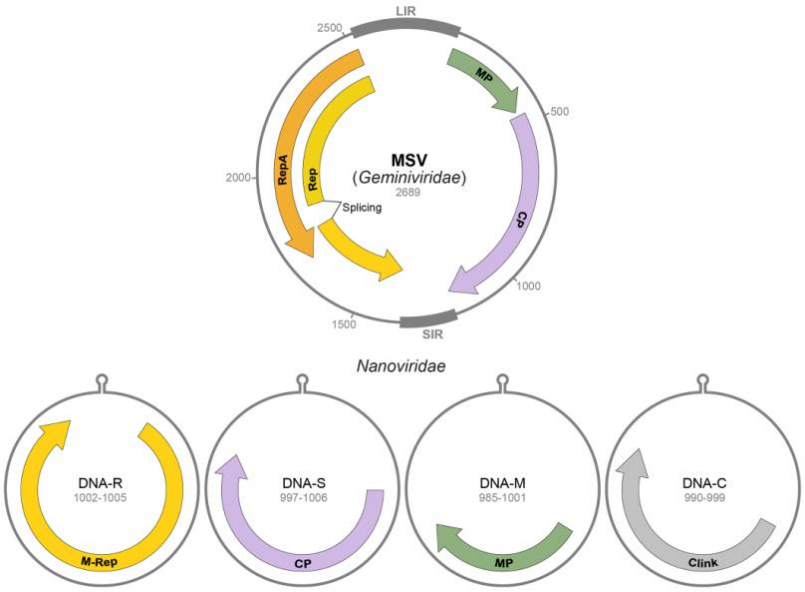
Comparison of plant virus DNA genomes encoding small hydrophobic MPs. Genes are shown as arrows with the names of the encoded proteins. MSV, *Maize streak virus*. LIR–long intergenic region; SIR – short intergenic region. For *Nanoviridae*, only four DNA components (DNA-R, -S, -M and -C) encoding the functionally characterized proteins are shown, and hairpin marks replication origin. The numbers indicate the sizes of circular genomic DNAs.

Forty years ago single-stranded DNA (ssDNA) plant viruses were thought to have an exclusive genomic architecture for DNA viruses adopted to plants because of their small sizes allowing passage through PD [193],[194]. However, more recent data have revealed that small circular ssDNA viruses encoding a homologous replication-associated protein (Rep) (Figure 2) are capable of replicating in the majority of eukaryotic multicellular and single cell organisms. Over the last decade, a considerable increasae in the number of discovered circular Rep-encoding ssDNA viruses (CRESS DNA viruses) allowed to delineate them as a special virus phylum Cressdnaviricota [195]–[196]. Currently, plant CRESS DNA viruses are included into two families *Geminiviridae* and *Nanoviridae* [197]–[199].

### 9.1. Geminiviruses

The viruses of the family *Geminiviridae* contain monopartite or bipartite DNA genomes with coding regions being located in both virion-sense and complementary-sense strands. The family *Geminiviridae* is one of the largest families of plant viruses. Small, single-stranded DNA genomes of geminiviruses encode 5–7 proteins that redirect host machineries and processes to establish a productive infection. Transcription of geminivirus genomes is bi-directional, and mRNA synthesis is initiated within the long intergenic region (LIR) (Figure 2). Multiple overlapping transcripts are used by geminiviruses for gene expression, and primary transcript splicing is also used by members of the genus *Mastrevirus* and probably *Capulavirus* [193],[197],[198],[200]–[202]. Among the best-characterized genomes of geminiviruses are those of members of genus *Mastrevirus* [197]. V1 (MP) and V2 (CP) ORFs are expressed from transcripts of the virion sense, while C1 and C2 ORFs are expressed from transcripts in the complementary sense. In contrast to CP and MP ORFs expressing from anti-sense transcripts, Rep is expressed from alternatively spliced sense transcript. MSV genome contains two intergenic regions, a smaller one (SIR) and a larger one (LIR) (Figure 2). The MSV MP is a small hydrophobic protein. In monopartite mastreviruses, the MP performs movement function in concert with CP, which is capable of nucleo-cytoplasmic shuttling and transport of viral DNA from the nucleus (site of replication) to cytoplasm, therefore being functionally equivalent to Nuclear Shuttle Proteins (NSP) of bipartite geminiviruses. Thus, the CP of monopartite geminiviruses can be regarded as an essential component in viral movement system [197],[198],[200]–[202]. It should be noted that putative MPs containing TMDs are also found in genus *Capulavirus* and some unclassified monopartite geminiviruses (Table S6).

### 9.2. Family Nanoviridae

The viruses of family *Nanoviridae* (genera *Nanovirus* and *Babuvirus*) contain multipartite circular ssDNA genomes composed of 6 to 8 segments of about 1kb. The ssDNA segments have a common stem-loop region and are encapsidated in separate particles (Figure 2) [194],[199]. One of the genomic components encodes a protein structurally and functionally homologous to geminivirus Rep protein. Another genomic segment encodes a small hydrophobic protein, which is found to localize exclusively to the ER and cell periphery [203]–[205]. Importantly, this TMD-containing protein (Table S6) is able to re-locate the NSP protein to the cell periphery. Thus, these results indicate that nanoviruses may utilize a system functionally similar to that of the bipartite geminiviruses, where the NSP protein acts to transfer ssDNA from nucleus to cytoplasm while the small hydrophobic MP protein transports the NSP-DNA complexes to the cell periphery for intercellular transport [204]–[206].

## 10. Conclusion

Association of plant virus replication and cell-to-cell transport with cell endomembranes dictate the necessity for viruses to encode hydrophobic proteins enabling functional interaction of viral complexes involved in replication and movement with membrane structures and, often, in remodeling of cell membranes for the formation of virus-specific replication/movement membrane structures. Therefore, comparative analysis of plant virus-encoded hydrophobic proteins and elucidating their evolutionary links may shed a new light on basic questions related to the possible common activities and structural peculiarities allowing them to serve as movement proteins. Particularly, are the small hydrophobic MPs always responsible for PD targeting, and what are the specific peculiarities of their transmembrane domains related to this targeting? If consider whole diversity of small MPs, it is evident that the wide ranges of the transmembrane domain sizes and very low similarity of their hydrophilic segments makes this task quite uneasy. However, our very recent studies of BMB2 and TGB2 MPs representing integral ER proteins open a new field for potential comparative experimental analysis. Constrictions of ER tubules upon high-level expression of reticulons resulted from the ability of reticulons to generate membrane curvature. Our results are consistent with the hypothesis that BMB2 and TGB2 may use a similar mechanism to induce membrane curvature [207]. We proposed a model that the hydrophobic segments of these proteins participate in the induction of ER constrictions and, thus, contribute to the TGB2- and BMB2-dependent increase in PD permeability and facilitating the virus intercellular spread. We hypothesize that this model can be applied to a broader range of plant virus small hydrophobic proteins [207].
