# Supplementary material for: Small hydrophobic viral proteins involved in intercellular movement of diverse plant virus genomes
Source: AIMS Microbiol. 2020 Sep 21;6(3):305–29. doi: 10.3934/microbiol.2020019 (PMC7595835; doi:10.3934/microbiol.2020019)
Supplement: Supplementary file 1 [file microbiol-06-03-019-s001.pdf]

AIMS Microbiology

AIMS Microbiology, 6 (3): 305–329.
DOI: 10.3934/microbiol.2020019
Received: 23 July 2020
Accepted: 13 September 2020
Published: 21 September 2020

http://www.aimspress.com/journal/microbiology

Review

Small hydrophobic viral proteins involved in intercellular movement of diverse plant virus genomes

Sergey Y. Morozov[1, 2, *] and Andrey G. Solovyev [1, 2, 3]

[1] A. N. Belozersky Institute of Physico–Chemical Biology, Moscow State University, Moscow, Russia
[2] Department of Virology, Biological Faculty, Moscow State University, Moscow, Russia
[3] Institute of Molecular Medicine, Sechenov First Moscow State Medical University, Moscow, Russia

* **Correspondence:** E–mail: morozov@genebee.msu.su; Tel: +74959393198.

Table S1. Hydrophobic motif comparisons among the small hydrophobic proteins encoded by some virus genomes in family *Closteroviridae*.

Virus name and motif position	Motif sequence	Proportion of hydrophobic residues	Charged residues	accession number
Citrus tristeza closterovirus (1–40)	MDCVIQGFLTFLVGIAVFCAFAGLIIIVITIYRCTIKPVR	29/40	4/40	NC_001661
Beet yellows closterovirus(1–38)	MDCVLRSYLLLAFGFWICLFLFCLVVFIWFVYKQILFR	31/38	4/38	AF056575
Beet yellow stunt closterovirus (1–39)	MDCILRAFLPFGFALVICFFIAVAAYFFAFFVKNTHSQD	30/39	4/39	BYU51931
Carnation necrotic fleck closterovirus (1–40)	MDCVLRSYLLLAFGFLICLFLFCLVVFVWFVYRNVLSVTP	32/40	3/40	GU234167
Carrot yellow leaf closterovirus (1–41)	MDSTLKFLLLFLFCWVCLIFSIVTFICIYLGISTFFYRLPD	31/41	4/41	FJ869862
Grapevine leafroll–associated closterovirus 2 (1–36)	MNQVLQFECLFLLNLAVFAVTFIFILLVFRVIKSFR	26/36	4/36	JX559644
Mint closterovirus 1 (1–33)	MDCTLRAYFYLLLGWIIVCFSFTLGFVVYKLVR	25/33	4/33	AY792620
Raspberry mottle closterovirus (22–60)	RSTRVPKSSILAFSLLLMLTLCLISAFFISCFRFHRFCR	25/39	6/39	NC_008585
Strawberry chlorotic fleck associated closterovirus (1–40)	MNCYERSDLDLLLGCATCAIIVFLVFICMYAYSTRPTFNK	27/40	6/40	DQ860839
Fig mild mottle–associated closterovirus (1–39)	MDCVIQGYLTLLMGCFVFLFKAFIAYVLISFRHLVGLPK	29/39	4/39	FJ611959
Grapevine leafroll–associated ampelovirus 1 (1–34)	MDLRQFSHELLYTVSLFILVALCFVVYFIVRAIR	25/34	5/34	JQ023131
Grapevine leafroll–associated ampelovirus 3 (1–43)	MDDFKQAILLLVVDFVFVIILLLVITFVVPRLQQSSTINTGLR	27/43	6/43	KJ174518
Little cherry ampelovirus 2 (1–40)	MSSSIWQCSAFSFILALLFAVLLSSSATVLVIYIIVPQIK	28/40	1/40	AF531505
Pineapple mealybug wilt–associated ampelovirus 2 (1–42)	MLDAFTAITIIASLILAFLFLLILFIVVLVYNYYSRMHSSMR	32/42	3/42	AF283103
Grapevine leafroll–associated ampelovirus 4 (1–40)	MLDLFSQFNWVFQICAFILIILFFAVLALVVQKVFYSTIR	31/40	3/40	NC_016416
Pineapple mealybug wilt–associated ampelovirus 1 (1–37)	MLRVDNFLWAIYLITFIALCAIIIVLILLFQRVLWPN	30/37	3/37	AF414119

Continued on next page

Virus name and motif position	Motif sequence	Proportion of hydrophobic residues	Charged residues	accession number
Pineapple mealybug wilt–associated ampelovirus 3 (1–37)	MIRVDNFLWAIYLLTFITISAIIVVLVVLIQRLLCPT	28/37	3/37	DQ399259
Plum bark necrosis stem pitting–associated ampelovirus (1–42)	MSQTLLATDISQVLLVFLIAFCFLIFITASLGVGFCVYRLIR	29/42	3/42	EF546442
Cordyline velarivirus 1 (1–31)	MIFLFFLFFLLFLFLLNLCTSPSNKGFIVHK	23/31	2/31	HM588723
Grapevine leafroll–associated velarivirus 7 (1–31)	MGILYEICVYLLVCAYIILKRPSEDELTFFK	21/31	6/31	HE588185
Little cherry velarivirus 1 (1–31)	MVVLVWVCLISVVCFFVSPSNIGLEKFSRFG	21/31	3/31	Y10237
Mint vein banding–associated velarivirus (1–37)	MVYGCGLSSSVLGDVLILISIVFIFVSAGFAFGRYTK	23/37	3/37	KJ572575
Olive leaf yellowing associated velarivirus (1–36)	MWPELRFTLCVLVSTLSFVFLACLILLIVHFSEIIK	27/36	4/36	AJ440010
Bean yellow disorder crinivirus (11–49)	FKICVWRNPLHILFDFLSIVNTLNLTASIYIDLVITWEV	26/39	5/39	EU191905
Beet pseudo–yellows crinivirus (22–60)	GLFDVPIRPHKNIYEISGFFNMLILCVLIFLFLNRFLLR	28/39	6/39	AY330919
Blackberry yellow vein–associated crinivirus (1–43)	MRLSLVCLLVSSRPFWSVSPLVFTFFCFVFFPFSHFSLFLLFE	33/43	3/43	AY776335
Cucurbit yellow stunting disorder crinivirus (1–41)	MYKNFSTIIFQFVCSRVKLWRRLVWILVLLSLLSAVMLMVL	29/41	5/41	AY242078
Cucurbit chlorotic yellows crinivirus (1–43)	MPKAFKFDRYLISLQPMSGSASSKNQHVSNNIYFSFCSIYLLS	24/43	5/43	AB523789
Lettuce chlorosis crinivirus p5.6 (6–50)	RMVAAFVSHHHPWPRVVYGAYLTMAGDESPSSQLPLSVRPVYPPP	32/45	5/45	FJ380119
Lettuce chlorosis crinivirus p6 (8–53)	MPKALKPYPSHWLSSLDIVILMRDLFITLDNSELDSVVCEQIHLTL	26/46	9/46	FJ380119
Potato yellow vein crinivirus RNA1 (16–61)	VGECGEDEVFRDRFSYIMIVYCYVLMVVLAVALCLIPCSKAIKQFL	32/46	9/46	AJ557128

Motif position means coordinates of the sequences presented in the table. GxxxG–like sequence motifs are shown in yellow. Transmembrane segments predicted by (http://www.cbs.dtu.dk/services/ TMHMM–2.0/) are underlined. Note that A, H, P and C residues are often regarded as hydrophobic in TMD segments.

Table S2. Hydrophobic motif comparisons among the TGB2 and TGB3 proteins encoded by some virus genomes in families *Alphaflexiviridae* and *Betaflexiviridae*.

Virus name and motif position	Motif sequence	Proportion of hydrophobic residues	Charged residues	accession number
TGB3 Potato virus X (1–50)	MEVNTYLNAIILVLVVTIIAVISTSLVRTEPCVIKITGESITVLACKLDA	31/50	7/50	YP_002332932
TGB2 Potato virus X (14–102)	SEKVYIVLGLSFALVSITFLLSRNSLPHVGDNIHSLPHGGAYRDGTKVILYNSPNLGSRVSLHNGKNAAFAAVLLLTLLIYGSKHISQR	49/89	11/89	AIL49076
TGB3 Bamboo mosaic potexvirus (1–52)	MLNTDTLCIILFILILGILYNILQQHLPPPCEIIINGHTISIRGNCYHTTSS	32/52	3/52	NP_042586
TGB2 Bamboo mosaic potexvirus (14–103)	TKAYLVLAIGVASALFLYTLTRNTLPHTGDNIHHLPHGGRYVDGTKGILYNSPTSSYPSSSLPFSMVIALATTLFLITKTILNPAPTTPR	51/90	8/90	BBG06251
TGB3 Foxtail mosaic virus (1–52)	MHESHLVVILALLLIALWCLSTRPVQPSCHVEINGHSIIVTGNCWHSTQRPH	33/52	4/52	NP_040991
TGB2 Foxtail mosaic virus (1–52)	DNTKAILTVAIGIAASLVFFMLTRNNLPHVGDNIHSLPHGGSYIDGTKSINYRPPASRYPSSNLLAFAPPILAAVLFFLTQPYLATRRS	53/89	10/89	AWT40558
TGB3 Papaya mild mottle associated virus (1–59)	MAILEYVQGVAARSASLSIVAIALLVYLILICLLSPQRPCEIVLTGESISIRNCEMSRD	37/59	9/59	QIJ97075
TGB2 Papaya mild mottle associated virus (8–102)	DYSRVYIVLSIGLTLGLISFCLTRYSLPVAGDFQHRFPFGGCYRDGNKLATYLPYQSTPQPYQLKFGQFEVISLIIIIVTTLIILGNGNPRICSR	53/95	12/95	QIJ97098
TGB3 Alfalfa virus S (1–74)	MQAPELVHTHASTSCQQSPWYSSPWALLLVSGLSALLVLAVADYFNNLPRSHSCLLTITGHSVSISGCENHDVP	44/74	5/74	QIC35025

Continued on next page

Virus name and motif position	Motif sequence	Proportion of hydrophobic residues	Charged residues	accession number
TGB2 Alfalfa virus S (8–93)	DYTKVFLAATIGAALALTTLFATRNTAPHVGDNIHHLPHGGLYQDGNKRIAYAGPGTGAHSRQHFLPAIAVVLLTLGIIISERFKR	53/86	12/86	QJD13459
TGB3 Vanilla latent virus (1–60)	MSYSLSSYFQPQYILVAIVALGLSYTALTVTGNFLKPANCIIEITGHSVVVSNCPTDQIP	36/60	3/60	YP_009389476
TGB2 Vanilla latent virus (8–90)	DHSKTYTALAIGAGAAVILFVLRQNTLPHVGDNIHHLPHGGCYQDGNKRITYGRLGNTSTHSWHVLLLIFLLSAAIYISSHRRFRVELHCAHCHR	53/95	13/95	ASJ78779
TGB3 Shallot virus X (16–69)	LRSPWQYFSLAVALAAVFAYAVLNLINGTTQGCLITITGSTTQISNCPLEHIP	32/53	2/53	Reference [129]
TGB2 Shallot virus X (8–92)	DYSKIYLALGCGLGLGFVVYASRVNHLPHVGDNTHNLPHGGQYCDGNKRVLYSGPKSGSSPTNNLWPFITVIALTLAILLTSCPRR	47/86	10/86	NP_620650
TGB3 Lolium latent virus (1–53)	MSLSFSLIVFAVGVAVSIGVLTLTTQQSSSYCLILVDGAKAVVEGCHLRQDIP	31/53	5/53	ACA53377
TGB2 Lolium latent virus (15–103)	DSLKQVYLTLAAGFAVGLGIFLLRTNTLPHTGDNIHHLPHGGCYRDGTKSIRYNSPGVATSSNIFLPAVAVLCILALLHVPFFQPDRVR	53/89	11/89	YP_001718501
TGB3 Indian citrus ringspot virus (1–49)	MHYIDWVILLTFAAALIVCLTPKPEPCIITVSGASATVSNCPNPELLTD	33/49	5/49	NP_203556
TGB2 Indian citrus ringspot virus (9–100)	HTWAVRIIALGLAVTALIFTSTRDTSRHVGDPSHSLPFGGHYRDGSKVIHYNSPRSSKPSNHTPYLLFAPIGIILLIHALHRLGNSAHICRC	54/92	12/92	NP_203555
TGB3 Potato virus M (1–44)	MIVHALIGLCAFCVVLFIITQNQSDCIVLITGESVRVQGCRIDK	27/44	6/44	QBL75479
TGB2 Potato virus M (8–98)	DFTKVYLSAALGVSLALVTWLLTKSTLPVVGDRDHNLPHGGWYRDGTKSVFYNSPGRLNSIEARKAPLLGQPWAIVVLLVLLIWASHKLGR	52/91	14/91	QBL75490

Continued on next page

Virus name and motif position	Motif sequence	Proportion of hydrophobic residues	Charged residues	accession number
TGB3 Hop latent virus (1–49)	MLTYLLACLVSCGLFLWLLNVSNPNQCLVILTGESVRVQGCVINEEFGR	29/49	4/49	AJR19306
TGB2 Hop latent virus (20–93)	RSDFHLCVMISLQVHRPVGVGRSSYARRRRAKLVGRCHRCY RLWPPTAFTTRCDNKTCFPGLTYNASIARFIRD	39/74	18/74	NP_066263
TGB3 Sweet potato chlorotic fleck virus (1–53)	MPPLWVTALIGFLLCFMTVVYIDSVRVVPSNCVIVITKSSITIRSCEKVPDLS	33/53	7/53	YP_164261
TGB2 Sweet potato chlorotic fleck virus (8–98)	DYSKSVLAFSVGCGIAVIIFVTTRSTLPYVGDGQHSLPHGGTYCDAAKRVIYGKPSRGSFDWLYTSGSASYAIPLILC LTVLIYCLSPKPQ	52/91	11/91	AMR69110
TGB3 Apple stem pitting virus (1–57)	MFPRSGLGLAVAAAVVAYLVLLLAQQLYMSNSSQCTIVITGESVSVVGCVYSEAFIE	36/57	4/57	NP_604467
TGB2 Apple stem pitting virus (1–57)	DYSKSVFPIAVGIAVAVVLFTLTRSTLPQVGDNIHNLPHGGNYQDGTKRISYCGPRDSFPSSSLISSGTPMIIGIIIF LIFAIYVSEKWSR	48/91	12/91	NP_604466
TGB3 Rubus canadensis virus 1 (1–51)	MLQINLVVVVLVSVFVVLVLTIIDKFERENPCFIQITGESVVIKGCLFDKD	31/51	10/51	YP_0069058 64
TGB2 Rubus canadensis virus 1 (8–98)	DYTGAAISVVIGLCIAFAFHSLTRSNLPHAGDNIHHLPHGGFYKDGTKVVAYGGPQSRFPSSNLFSSSFSSLSVLCVI LLLSGLIYASNKF	51/91	8/91	YP_0069058 63
TGB3 Cherry necrotic rusty mottle virus (1–50)	MRAVDLLIGLLVFSITAYTIGLFNFSSSNSCTVIITGEKAVVTGCEITPE	27/50	6/50	BAU25808
TGB2 Cherry necrotic rusty mottle virus (8–101)	DYSRPLLCAVIGVSLAVICASFKANYLPSVGDNLHSLPHGGSYRDGTKAINYNGLNCVSGTSVDSQFLPSKFVAFSFV CIISLLIYACSRPTHR	54/94	11/94	BAU25807

Continued on next page

Virus name and motif position	Motif sequence	Proportion of hydrophobic residues	Charged residues	accession number
TGB3 African oil palm ringspot virus (1–54)	MSPEVALLVVVIFTIATVIFTELFSSRGALGSQKGCYILVTGERALVSGCELNK	30/54	8/54	YP_0027763 50
TGB2 African oil palm ringspot virus (3–99)	RPPTNYTNAILAASIGVAVGLLIHFVRRNELPHSGDNIHHLPYGGQYMDGTKCINYNRGFGGRNFSLLGTSSNSGIWL LLLTTGLLLYVTRSCFAR	50/96	11/96	YP_0027763 49

Motif position means coordinates of the sequences presented in the table, and proportions are related only to the sequences included into the table. GxxxG–like sequence motifs are shown in yellow. Transmembrane segments predicted by (http://www.cbs.dtu.dk/services/TMHMM–2.0/) are underlined. Note that A, H, P and C residues are often regarded as hydrophobic in TMD segments.

Table S3. Hydrophobic motif comparisons among the TGB2 and TGB3 proteins encoded by some virus genomes in family *Virgaviridae*.

Virus name and motif position	Motif sequence	Proportion of hydrophobic residues	Charged residues	accession number
TGB2 Barley stripe mosaic virus (11–97)	KYWPIVAGIGVVGLFAYLIFSNQKHSTESGDNIHKFANGGSYRDGSKSISYNRNHPFAYGNASSPGMLLPAMLTIIGIISYLWRTRD	45/87	11/87	AAA79162
TGB3 Barley stripe mosaic virus (52–155)	DILDDHYYAILASLFIIALWLLYIYLSSIPTETGPYFYQDLNSVKIYGIGATNPEVIAAIHHWQKYPFGESPMWGGLVSVLSVLLKPLTLVFALSFFLLLSSKR	67/104	12/104	AAA79163
TGB2 Lychnis ringspot virus (11–104)	KYWPVVVGVGLVSLFAYLIFTNQKHATQSGDNIHKFANGGFYQDGNKRINYNKNNNLAYGYRGLSNASSTELWMLGLCAAAIAAGVYGEYLRKR	48/94	14/94	AZL87783
TGB3 Lychnis ringspot virus (29–157)	DNRMSATPVLQTAKSVVGGDNFFLVFYSFIAGILLALLFVWLVGGTTCSLPQRASYYYQDLNKVEIEVVPGSPIDPEVIKAIHHFQRFPYGKTPGFGWLEDLTTLVSVWFTRLLYLLIIFFLAWIFKNI	77/129	18/129	AZL87782
TGB2 Beet virus Q (9–104)	RPNIYWPIVVGVVAIALFGFLTITNQKHSTQSGDNIHKFANGGSYADGSKRINYNKNNCRAYNGSSSNRTFTGLLLPALFLAAALYAYVCWSKPK	50/95	12/95	NP_612612
TGB3 Beet virus Q (44–180)	REESSFSLSYVALCCVVCLLLGVTFSLYLKSGAEVDSSAFSYYYQDLNSVEVKIGSYPIDPEIIKAIHHFQEAPFGVSLSQSDDSDVDDVPDVAELALQIDRLTLSCVVFEFIEKLCYRFFCVCLVVFCFYCYFHFC	84/137	26/137	NP_612613
TGB2 Broad bean necrosis virus (11–105)	PNKYWPILFGVSAICFFLFLGVTNQNIPHNHHGDNIHKFSNGGKYQDGTKRINYNANNSRAYNGSSSNNQFKGLFLPALLFTAAMLAFQWFSKSR	48/95	11/95	BAA34697
TGB3 Broad bean necrosis virus (48–176)	RESVFDSYYLLICCSVCFILGLSIMLFINNLYFRNVSSVGGSYYYQDLNSVEYKSSGPIDADVIERIHHFQQGPLGRFKDDATFAIKVKEDDFFEDAVDIRETSLFISDKVVSFVFIIILILLLKVCYG	72/129	28/129	BAA34698
TGB2 Gentian ovary ringspot virus (8–101)	RPNKAWPVVIGIAIVGLFLYLGSTHQKHATSSGDNIHKFSNGGTYRDGTKSISYNKNNNRAYNNGSSGDRTSAGLLLLLLCTTCCVWIHFQAKK	43/94	14/94	BAP18646

Continued on next page

Virus name and motif position	Motif sequence	Proportion of hydrophobic residues	Charged residues	accession number
TGB3 Gentian ovary ringspot virus (46–153)	EGVVFKTCLLLAIFIFTFGFYSFFFAGDSTELNNKGVSIYYQDLNMVEIRQYPGNEISPELIRQIHHFQKRPFGLPSDSLFDA WCPDVMEFAIFILGLVLIFLIMRTC	64/108	18/108	BAP18647
TGB2 Drakaea virus A (10–100)	RPNKVWPLVVGVCVIALFLFLSVVNKKHATESGDNIHKFANGGTYRDGSKCISYNRNHNLAYGGSSSNTTFLKLFLPILL VVAITILSRVR	48/91	14/91	YP_0096659 77
TGB3 Drakaea virus A (42–149)	PKLSHYLMIFLVAFSGSLMTVLLLYVCGIFDKHCDASYSYYYQDLNSVEFKSVPGNPIDPETVKAIHHFQKFPFGLSPMFS MFISIFQSLSVPGFIMILCVCLFKIAH	72/108	11/108	YP_0096659 78
TGB2 Peanut clump virus (11–101)	NKYWPGVVAIGLVSLFIFLSVSNQKHSTTSGDNIHKFSNGGTYRDGSKCITYNRNSPLAYNGSSSNNTLFWLCLLGLSMV WIAYCGYKSLS	45/91	9/91	NP_620031
TGB3 Peanut clump virus (41–153)	KTRETSFLSVLNDNAWLFVIAALILCLYFIISKPHVDAVYTEFHQDLNGFSMKLAPGVPIDPKVIAAVKNWQKYPFGTDP RENMVTSIVSGLRHSFCILLLVVVLLVYVCHKP	71/113	18/113	NP_620032
TGB2 Indian peanut clump virus (11–100)	NKYWPGVVAIGLVTLFVFLSVSNQKHSTTSGDNIHKYANGGTYRDGSKCISYNKNSPLAYNGSSSNNTLFWLCLLGLSM VWIAFCGYKSL	45/90	9/90	NP_835266
TGB3 Indian peanut clump virus (43–155)	KETTFLSVLNDNLWLFVVAVGILCVVFLSVWCETHCGACLCLSFTKNLNGVSIKVAPGAPIDPNVIAAIHHWQKYPFGE NPNAKIVVSVIDSIKRGLCMLLLCVTLLLYVCYK	74/113	14/113	NP_835267

Motif position means coordinates of the sequences presented in the table, and proportions are related only to the sequences included into the table. GxxxG–like sequence motifs are shown in yellow. Transmembrane segments predicted by (http://www.cbs.dtu.dk/services/TMHMM–2.0/) are underlined. Note that A, H, P and C residues are often regarded as hydrophobic in TMD segments.

Table S4. Hydrophobic motif comparisons among small hydrophobic movement proteins encoded by virus genomes in the family *Benyviridae* and BMB–containing viruses.

Virus name and motif position	Motif sequence	Proportion of hydrophobic residues	Charged residues	accession number
TGB2 Beet necrotic yellow vein virus (1–106)	MSREITARPNKNVPIVVGVCVVAFFVLLAFMQQKHKTHSGGDYGVPTFSNGGKYRDGTRSADFNSNNHRAYGCGG SGGSVSSRVGQQLVVLAIVSVLIVSLLQRLR	49/106	16/106	NP_612618
TGB3 Beet necrotic yellow vein virus (1–132)	MVLVVKVDLSNIVLYIVAGCVVVSMLYSPFFSNDVKASSYAGAVFKGSGCIMDRNSFAQFGSCDIPKHVAESITKVA TKEHDADIMVKRGEVTVRVVTLTETLFIILSRLFGLAVFLFMICLMSIVWFWCHR	82/132	21/132	NP_612619
TGB2 Beet soil–borne mosaic virus (11–118)	KNVPIVVGVCVVAFFVLLAFMQQKHKTHSGGDYGVPTFSNGGKYRDGTRSADFNSNNHRAYGCGGSKSSVTGKV GQQLLVLALVVAVFVLFMRGCWSSPEHICNGSCG	54/108	14/108	YP_009513204
TGB3 Beet soil–borne mosaic virus (1–132)	MVLVVKVDFSTIVLYIVAGVVVVSVLYSPFFSNEVKAGGYAGAIFPNGGCIMDRNSFAQFGGCDIPKYVADSISRVAI KELDADIKADLNSVVAKRVVLYEGLAQLCYRVFSWLVCLFMVCLMLFVWFWYHS	84/132	20/132	YP_009513205
TGB2 Burdock mottle virus (11–110)	NTKYIVLGVCVVAFICFLGFSQQKHATHSGDGVGVPRFANGGSYRDGTRSMNFNSNNPNAYGCKSEGFFGGFEKLA LLFLVLGIILYVAGGCAGGDHVCN	50/100	12/100	YP_008219067
TGB3 Burdock mottle virus (6–114)	RFDVTLCVLYIVCGIVVVCVVHSPVFQHPPPVSRVGDAVFLGDGYYSDQHATVVFGNFDASRVNTEHISSIAKSEHL VDIVGSMRSFAGDIAPTLVVICLVLLLGQRIK	67/109	15/109	YP_008219068
TGB2 Rice stripe necrosis virus (11–110)	NKFIPICICIGVVCVAVCLVLATPRHKTHSAGDYGVPTFANGGSYADGTRRAKFNCNNDRAYGSSQPQMSSNFVAFI AVILLVAFALRSCNSVGNCGERC	56/100	13/100	QJE38064
TGB3 Rice stripe necrosis virus (8–133)	DWSEIVLYVTGGIVVVTMVFLWSTVQRHEPPPGVAGAMPYGGLELNRASLNVYNHPSFDSAAAIKEMGKIADALV LMNRDDSIMMAGLSPIYGGFQVVCNIISRVFWLVVVIFWFIVVPCWFWCHK	81/126	16/126	QJE38065
TGB2 Nicotiana velutina mosaic virus (14–107)	DKTWLYLAGVSVLGLYVLVGYLTTSPKWRTASGGDYMVPTFANGGTYRDGTRMVSFNSNTGRFPWAINSRSSLVD VVVIILIISVILLHKFSGE	49/94	13/94	BAA00754

Continued on next page

Virus name and motif position	Motif sequence	Proportion of hydrophobic residues	Charged residues	accession number
TGB3 (partial) Nicotiana velutina mosaic virus (8–102)	DVSKIVCYISWVVGAIVVAYMYTHSVSDNGITHGNNNSRNIVMNALMLEGTEQVDSELLGVVASEIGYTYRVVVLCDTLVSVVSNIVTIIVLVIS	54/95	11/95	BAA20965
TGB2 Colobanthus quitensis VLRA (9–105)	RADYTPLFVIVGGVLILSGLFMFKTPERIHSGGDQGVASFRNGGSYTDARKAVFNPVHKVHDPSNSKVSAAGAEAILLVTSVVICLILKWINGQVQ	53/96	15/96	GCIB01126289
BMB2 Hibiscus green spot virus (1–87)	MPIFQGQAKSNAPVLFVCASICVIAYFFFNPGNPYNVPLSPGHVFPFGGEYAVHARFNGPSFPLSMGGSSVGLLMLCVILLVVVTHM	59/87	3/87	YP_004928124
BMB2 Lathyrus sativus VLRA (11–100)	DDTKVIIVYGFMVLIGCCFFIYNSVNPGNPYNVGLAPGHVFPHGGRLIVTAEYNSPPGWFQFKECPSLNFLLMFVMALITVIFFSPRLE	58/89	9/89	YP_004928123
BMB2 Litchi chinensis VLRA (1–92)	MAILNAPNNVSLNLNIVCVFCCVCLCFICWCMNNPGNPYDVGFANGFVFPNGGRYMVQAEFLPPKFSLFNFRGNGSFSLVLICVGILVYLRF	61/92	6/92	WAXR–2010981

Motif position means coordinates of the sequences presented in the table, and proportions are related only to the sequences included into the table. GxxxG–like sequence motifs are shown in yellow. Transmembrane segments predicted by (http://www.cbs.dtu.dk/services/ TMHMM–2.0/) are underlined. Note that A, H, P and C residues are often regarded as hydrophobic in TMD segments.

Table S5. Hydrophobic motif comparisons among small hydrophobic movement proteins encoded by genomes of DGB–containing viruses and members of family *Luteoviridae*

Virus name and motif position	Motif sequence	Proportion of hydrophobic residues	Charged residues	accession number
DGB 2 Carnation mottle virus (1–60)	MPSVNLHLIVLTGVIGLMLLIRLRCTFTSTFSLPPLVTLNQIIALSFCGLLLNSISRAER	36/60	5/60	BBM06286
DGB 2 Pelargonium flower break virus (19–100)	RYPSQRTLSPNGHLMVVMGVLGLLWLRPFRSIYTSTFSMPPL INLQHILNLTLLSLILSSFILAERVTHNHYSNDNSKAQYI	47/82	8/82	NP_945126
DGB2 Saguaro cactus virus (1–59)	MLCGNKHLALLMGVIGLLLLIRWRFILPSTFSLPPLVTLEQIIVLSFCGLLISCACKAD	41/59	6/59	NP_044387
DGB2 Tobacco necrosis virus A (1–48)	MAYCRCCDTSPGITLFPYFAILILILAILVVGTPNQQYHHSPSTYEYK	43/94	43/94	AAT69240
DGB2 Olive latent virus 1 (1–48)	MAVCRCCDTSPGITLFPYFAILILILAILVVGTPNQQYHHSPSTYEYK	33/48	4/48	ADE48192
DGB2 Turnip crinkle virus (1–60)	MRILLVTGVLGLLLLIRLRSQSTSTFDNCQCPTSPWVIYAFYNSLSLVLLLCHLIPEIKP	38/60	6/60	QBG64839
DGB2 Hibiscus chlorotic ringspot virus (1–55)	MKLFLGVLSFLLLIKLRLPLTLTFDVDLRTLRENCFIITTLSLFGFFITVLIKAE	34/55	10/55	NP_619675
DGB2 Tobacco necrosis virus D (1–38)	MKYIIVQQNDPFPLLGVWIIVIIIIAVIGLLNQSPPER	26/38	4/38	ASA69345
DGB2 Leek white stripe virus (1–44)	MVIVVRESSFPLLAVWIIVIVLVAVIGALLQTPPETINQVFNED	30/44	5/44	NP_044743
DGB2 Galinsoga mosaic virus (1–48)	MKYCRCSDTAPTDHITLLFVIFILSGLILSLCTNITSNNYENHTTENK	24/48	6/48	NP_044735
DGB2 Melon necrotic spot virus (11–50)	GDYSGPLLILFLAFVFFYITSLGPHGNTYVHHFDNSSVKT	24/40	3/40	BAG13033
DGB2 Pea stem necrosis virus (9–35)	RDTLVPFLAIIICILLILISFLGQQER	18/27	4/27	NP_862838
DGB2 Furcraea necrotic streak virus (7–48)	DNEPTLSPLFPFFAILVLVLAILVVGTPHVQYQSDQHHNESK	26/42	5/42	YP_007517177
DGB2 Maize chlorotic mottle virus (7–49)	DSSWPQWLRNLILGILISSILFILTKTQDTVAVYHEPSVYSID	24/43	6/43	YP_009237216
DGB2 Panicum mosaic virus (11–51)	DPRVGPLLVLCLLLLLILFSRSWNVAPVVVPSYHTVYHHEK	30/41	5/41	AAC97554

Continued on next page

Virus name and motif position	Motif sequence	Proportion of hydrophobic residues	Charged residues	accession number
DGB2 Cocksfoot mild mosaic virus (10–52)	DNPFVWPLVTVVLILLIIIVASVTSTGPVIIPPSHNTTYHHEK	29/43	3/43	YP_002117837
DGB2 Pelargonium line pattern virus (1–76)	MEYPRVHLAILSVLISSQLLIKWNLWSISISDFLPQPHSLHPNLLVCIVLCIFFSSVLSQGQSYSYSYFSTSTSDK	45/76	6/76	AGZ62052
DGB2 Elderberry latent virus (5–72)	REHILTLLVLCLSLLLIKSHFTLPSISRPSFDSPNLLVSIVLGLFFASVLTPGNYVYSYYSANSTSDK	40/68	7/68	YP_009116637
P2 Ribes americanum virus A (1–39)	MNFKSYLLKKIKSVGIGLASSLIIYIASFVFNVLYRRSF	22/39	6/39	NC_040797
P3 Ribes americanum virus A (1–60)	MCYIDVAFDLVCLFICVLILVALLKLTYCNSSAFCVALALTIYSLFLNFNLLVLLYDLSR	43/94	43/94	NC_040797
P2 Rhododendron delavayi VLRA (1–61)	MLFLVLELLQIAVLIFLLVKCLALFTGSFARLSPGSRLSFWILWSACLVLIILICLRVGQD	45/60	5/60	GFCU01057973
P3 Rhododendron delavayi VLRA (1–31)	MCQELSCAFAVLLALSVISTVISVYLLIDCR	22/31	3/31	GFCU01057973
P3a Bean leafroll luteovirus (5–40)	DYKFLAAFVLGFVSNIPVTALGIYIVYQRILKEIR	23/35	6/35	NC_003369
P3a Soybean dwarf luteovirus (2–36)	DYKFLSGFASGFISSIPVSILAIYFVYLKISKNLR	21/35	5/35	NC_003056
P3a Barley yellow dwarf luteovirus–MAV (1–38)	TNINYQILAGFGIGFIASIPCTLALTYFIYNKVSDTTR	21/38	3/38	NC_003680
P3a Potato leafroll virus (2–36)	DYKFLAGFALGFSSAIPFSVAGLYFVYLKISSHVR	23/35	4/35	NC_001747
P3a Turnip yellows polerovirus (2–36)	DYKFLAGFAAGFVSSIPISVISIYFIYLRISKHVR	23/35	5/35	NV_003743
P3a Melon aphid–borne yellows polerovirus (5–40)	DFRFVAGFTSGFLAAIPLCALGLYLIYLKISAHVR	25/35	4/35	NC_010809

Motif position means coordinates of the sequences presented in the table, and proportions are related only to the sequences included into the table. GxxxG–like sequence motifs are shown in yellow. Transmembrane segments predicted by (http://www.cbs.dtu.dk/services/ TMHMM–2.0/) are underlined. Note that A, H, P and C residues are often regarded as hydrophobic in TMD segments.

Table S6. Hydrophobic motif comparisons among small hydrophobic movement proteins encoded by plant virus DNA genomes.

Virus name and motif position	Motif sequence	Proportion of hydrophobic residues	Charged residues	accession number
Maize streak virus (27–60)	RVGEVAILSFVALICFYLLYLWVLRDLILVLKAR	26/34	6/34	YP_009154761
Chloris striate mosaic mastrevirus (38–82)	RSGSVEQPSPGASFAFPVKVTALVCFAAIVGACILVFLYKTCIAD	27/45	5/45	NP_040950
Miscanthus streak mastrevirus (12–42)	DDGALHGILVAFIAVLCLIGCLWAAYRLFLK	24/31	4/31	NP_569144
Tobacco yellow dwarf mastrevirus (36–74)	EHFFSKVVVALIVILFAVGIVYLAYTLFLKDLILLLKAK	30/39	6/39	NP_620724
Alfalfa leaf curl capulavirus (1–46)	MLWNMSLGSVFMEYCAAWIFWWQNIVLIFLFTILFWINGKSFILAR	32/46	3/46	AKQ51597
Euphorbia caput–medusae latent capulavirus (1–42)	MFSKRSLGHPSTVYGILCLLDILVAEYFINFYFFIYYFYKWK	29/42	6/42	CCV02655
Euphorbia caput–medusae latent capulavirus (1–36)	MEYCASLIFWWQNILLIFIFSFIIFINGKTFLLLRR	25/36	4/36	CCV02656
Plantago lanceolata latent capulavirus (1–36)	MEYCSEVLFWTYFLIITIYFALILYFNGKTLLLGRR	23/36	5/36	ANA76382
Mulberry crinkle–associated geminivirus (23–53)	DSKYCTTILIVQFIVIALAILSYVFVEYQIR	21/31	4/31	AKM94181
Camellia chlorotic dwarf–associated geminivirus (32–62)	ENKVLFALCILFIIYGLVCNVVFCYGGFTRK	21/31	4/31	NC_040817
Paper mulberry leaf curling associated geminivirus 2 (12–42)	REVLFFACVAIIFFSLVLNVSVFVKTAGFAR	21/31	4/31	QJX74418
Paper mulberry leaf curling associated geminivirus 1 (6–43)	EVEYCLSSSSLTGFFICISIFLLLLFCQYGYVAVQIRR	24/38	4/38	QJX74404
Banana bunchy top babuvirus (12–40)	FEWFLFFAAIFIAITILYILLVLLFEVPK	25/29	3/29	AFN27077
Abaca bunchy top babuvirus (12–40)	FEWFLFFSAIFVAITIIYILLAVLLELPK	24/29	3/29	YP_001661658
Cardamon bushy dwarf babuvirus (12–40)	FEWFLFFAAIFVAITIFYILLALLLEIPR	25/29	3/29	YP_009508037
ORF4 protein Coconut foliar decay virus (12–43)	DSIWIRTNLLLCLQCTQPLSTSPIQVSSLLEK	16/32	4/32	NP_040949
Subterranean clover stunt nanovirus (22–55)	EVLYKIGIIMLCIVGIVVLWVLIILCCAVPRYAK	26/34	4/34	QBF29186

Virus name and motif position	Motif sequence	Proportion of hydrophobic residues	Charged residues	accession number
Faba bean necrotic yellows nanovirus (20–57)	KRHQALYLIGIILLVTVCLTVLWVCIMLACYVPGFLKK	28/38	4/38	NP_619569
Milk vetch dwarf nanovirus (20–57)	KRHQALYLIGIIILIMVCIIILWVCIMLACYIPGFLKK	30/38	4/38	QJW28675
Pea yellow stunt nanovirus (20–57)	RHQVLYIIGIVLLIMLCIVVLWVCIMLACFLPGFLKR	30/37	3/37	YP_008997795

Motif position means coordinates of the sequences presented in the table, and proportions are related only to the sequences included into the table. GxxxG–like sequence motifs are shown in yellow. Transmembrane segments predicted by (http://www.cbs.dtu.dk/services/ TMHMM–2.0/) are underlined. Note that A, H, P and C residues are often regarded as hydrophobic in TMD segments.

AIMS Press

© 2020 the Author(s), licensee AIMS Press. This is an open access article distributed under the terms of the Creative Commons Attribution License (http://creativecommons.org/licenses/by/4.0)
